# Supplementary material for: The Impact of Iron Supplementation for Treating Anemia in Patients with Chronic Kidney Disease: Results from Pairwise and Network Meta-Analyses of Randomized Controlled Trials
Source: Pharmaceuticals (Basel). 2020 Apr 30;13(5):85. doi: 10.3390/ph13050085 (PMC7281268; doi:10.3390/ph13050085)
Supplement: Supplementary file 1 [file pharmaceuticals-13-00085-s001.pdf]

Supplementary data for the following article:
The impact of iron supplementation for treating anemia in patients with Chronic Kidney Disease: Results from pairwise and network meta-analyses

Marcel Adler, Francisco Herrera-Gómez, Débora Martín-García, Marie Gavid, F. Javier Álvarez, Carlos Ochoa-Sangrador

Table S1. Participants, interventions, comparators, and outcomes in (a) the trials that provided numerical data for the meta-analysis and (b) the trials that did not provide numerical data for the meta-analysis.

Table S2. Risk of bias in the eligible studies.

Figure S1. Inconsistency plots on random effects for the SUCRA assessable iron supplements values in the subgroups of (a) patients in the KDIGO GFR categories 3A to 5, and (b) dialysis patients.

Panel S1. Search strategy formulae and search results.

Table S1. Participants, interventions, comparators, and outcomes in
(**a**) The trials that provided numerical data for the meta-analysis.[&]

**Trial** Details	**Design**	**Follow-up** (in wks)	**Participants/population** Characteristics	**Interventions** (n)	**Comparators** (n)	**Outcomes**	**Co-interventions**
**FACT** NCT01227616 USA, Canada and UK [1].	RCT	55	M age in yrs (SD), males (%), DM (%):[$] 58.8 (14.0), 58.4, NA. KDIGO GFR G3a–G5/KRT (%): 0.0/100.0 Causes of CKD (%): NA.	Ferumoxytol IV 2 x 510 mg during 1 wk repeatable at the end of eleven 5-wk TPs (196).	Iron sucrose IV 10 x 100 mg in 10 HD sessions repeatable at the end of eleven 5-wk TPs (97).	Changes in Hgb and TSAT. ≥1.0 g/dL increase in Hgb. Intensification of ESA therapy and/or need of blood transfusions.[§] TEAE. Oxidative stress parameters. MRI-based iron load.	Fixed ESA dosing during each 5-wk TP, but adjustable dosing inter 5-wk TPs. Blood transfusions.
**PIVOTAL** EudraCT 2013-002267-25 UK [2].	RCT STR	168	M age in yrs (SD), males (%), DM (%):[$] 62.8 (15.0), 65.3, 44.4. KDIGO GFR G3a–G5/KRT (%): 0.0/100.0 Causes of CKD (%): DM (33.5), TIN/HTN (27.4), GN/AID (18.6), unknown/other (15.0), ADPKD (5.5).	Iron sucrose IV 400 mg/4 wks (1093).	Iron sucrose IV 100–200 mg/4 wks (1048).	MACE, HF, death. Intensification of ESA therapy and/or need of blood transfusions.[§] TEAE. Quality of life.	Adjustable ESA dosing. Blood transfusions.
**COSMOS study 2015** NCT01222884 India, UK, Russia, EU, Switzerland,	RCT STR	8	M age in yrs (SD), males (%), DM (%):[$] 59.9 (15.9), 65.8, 33.9. KDIGO GFR G3a–G5/KRT (%): 0.0/100.0 Causes of CKD (%): NA.	Iron isomaltoside IV 500 mg during the 1st wk (117). Iron isomaltoside IV	Iron sucrose IV 100 + 100 + 200 mg during 4 wks (117).	Hgb 9.5 to 12.5 g/dL. Changes in Hgb, SF, TSAT, Fe, and Ret. TEAE.	Fixed ESA dosing.

and USA [3].				100 + 200 + 200 mg during 4 wks (117).		Quality of life.	
**Princess Alexandra Hospital study** ACTRN12608000186358 Australia [4].	RCT	12	M age in yrs (SD), males (%), DM (%):[@] 46.4 (12.7), 73.0, 3.0. KDIGO GFR G3a–G5/KRT (%): 0.0/100.0 Causes of CKD (%): GN/AID (45.0), unknown/other (34.0), ADPKD (14.0), TIN/HTN (4.0), DM (3.0).	Iron polymaltose IV 500 mg during the 1st wk (51).	Iron sulphate PO 2 x 100 mg/d (51).	Hgb ≥11.0 g/dL. Initiation of ESA therapy and/or need of blood transfusions.[§] TEAE. Acute graft rejection.	Initiation of ESA if required (not all participants on ESA therapy). Blood transfusions. CNI-based immunosuppre-ssive therapy.
**Ferumoxytol authorization studies** NCT00233597 USA [5, 6].	RCT	5	M age in yrs (SD), males (%), DM (%):[$] 60.2 (13.7), 56.5, 42.6. KDIGO GFR G3a–G5/KRT (%): 0.0/100.0 Causes of CKD (%): DM (42.6), TIN/HTN (34.3), unknown/other (15.2), GN/AID (7.9).	Ferumoxytol IV 2 x 510 mg during the 1st wk (114).	Iron fumarate PO 4 x 50 mg/day for 3 wks (116).	Changes in Hgb, SF, TSAT, Fe, TIBC, and CHr. ≥1.0 g/dL increase in Hgb. TEAE.	Fixed ESA dosing (not all participants on ESA therapy).
**DRIVE & DRIVE II** NCT00224081 USA [7, 8].	RCT STR	12	M age in yrs (SD), males (%), DM (%):[$] 59.2 (14.7), 50.4, NA. KDIGO GFR G3a–G5/KRT (%): 0.0/100.0 Causes of CKD (%): NA.	Iron gluconate IV 8 x 125 mg in 8 HD sessions (68).	Non-iron (66).	Changes in Hgb, SF, TSAT, and CHr. ≥2.0 g/dL increase in Hgb. Change in CRP.	25% increase in ESA dosing at randomization.
**Beijing Chaoyang Hospital study** China [9–11].	RCT	12	M age in yrs (SD), males (%), DM (%):[$] 55.8 (14.2), 43.8, 14.7. KDIGO GFR G3a–G5/KRT (%): 0.0/100.0 Causes of CKD (%): DM (47.8), TIN/HTN (32.6), unknown/other (17.4), ADPKD (2.2).	Iron sucrose IV 200 mg/wk for 4 wks followed by 100 mg/wk for 8 wks (102).	Iron succinate PO 3 x 200 mg/d for 12 wks (92).	Changes in Hb, Hct, SF, TSAT, and Ret. ≥1.5 to 3.0 g/dL increase in Hgb or ≥5% to 10% in Hct.	Fixed ESA dosing.

**US Venofer CT study 2006** USA [12].	RCT STR	8	M age in yrs (SD), males (%), DM (%):[$] 54.0 (15.1), 56.6, NA. KDIGO GFR G3a–G5/KRT (%): 0.0/100.0 Causes of CKD (%): NA.	Iron sucrose IV 300 + 300 + 400 mg during 4 wks (80).	Non-iron (46).	Changes in Hgb, SF, TSAT, and Fe. ≥0.75-1.0 g/dL increase in Hgb. Intensification of ESA therapy, increase of iron supplement dosing and/or need of blood transfusions.[§] TEAE.	Adjustable ESA dosing. Blood transfusions.
**KfH Dialysis Centre study** Germany [13].	RCT	24	M age in yrs (SD), males (%), DM (%):[$] 59.0 (15.0), 44.0, NA. KDIGO GFR G3a–G5/KRT (%): 0.0/100.0 Causes of CKD (%): NA.	Iron sucrose IV 250 mg/4 wks (27).	Iron gluconate IV 62.5 mg/wk (28).	Changes in Hgb, SF, TSAT, and Fe. Intensification of ESA therapy.[§] TEAE.	Adjustable ESA dosing. Stop of iron supplement if Hgb ≥12.5 g/dL or SF ≥1000 µg/L.
**Barts study** UK [14].	RCT	16	M age in yrs (SD), males (%), DM (%):[$] 53.0 (13.7), 56.8, NA. KDIGO GFR G3a–G5/KRT (%): 0.0/100.0 Causes of CKD (%): NA.	Iron dextran IV 250 mg/2 wks (12).	Iron sulphate PO 3 x 200 mg/d (13). Non-iron (12).	Changes in Hgb, SF, TSAT, Fe, and TIBC. Intensification of ESA therapy.[§]	Scheduled adjustable ESA dosing.
**Winthrop University Hospital study** USA [15].	RCT	16	M age in yrs (SD), males (%), DM (%):[$] 49.5 (9.3), 59.6, NA. KDIGO GFR G3a–G5/KRT (%): 0.0/100.0 Causes of CKD (%): NA.	Iron dextran IV 2 x 100 mg/wk (20).	Iron sulphate PO 3 x 325 mg/d or iron polysaccharide PO 2 x 150 mg/d (36).	Changes in Hgb, SF and TSAT. Intensification of ESA therapy.[§]	Scheduled adjustable ESA dosing.
**KCH/RAH studies** NCT01052779 NCT01114204 Global [16–18].	RCT	5	M age in yrs (SD), males (%), DM (%):[€] 51.1 (15.9), 56.8, NA. KDIGO GFR G3a–G5/KRT (%): 15.4/10.4 Causes of CKD (%): NA.	Ferumoxytol IV 2 x 510 mg during the 1st wk (486).	Iron sucrose IV 10 x 100 mg in 10 HD sessions or 5 x 200 mg during 2 wks	Changes in Hgb, SF and TSAT. ≥1.0 to 2.0 g/dL increase in Hgb. Hgb ≥12.0 g/dL.	Adjustable ESA dosing (not all participants on ESA therapy). Blood

					(281).	Intensification of ESA therapy.[§]<br>TEAE.	transfusions.
**Luitpold Pharma study**<br>NCT00548691<br>USA [19].	RCT	4	M age in yrs (SD), males (%), DM (%):[¥] 60.2 (12.6), 39.2, NA.<br>KDIGO GFR G3a–G5/KRT (%): 18.9/81.1<br>Causes of CKD (%): NA.	FCM IV 750 to 1000 mg during the 1st wk or 200 mg into 1 HD session (258).	Various iron supplements therapy options (IV and PO) including non-iron option (259).	Changes in Hgb, SF and TSAT.<br>≥1.0 g/dL increase in Hgb.<br>Hgb ≥12.0 g/dL.<br>TEAE.	Fixed ESA dosing (not all participants on ESA therapy).
**COSMOS study 2016**<br>NCT01102413<br>India, UK, EU, and USA [20].	RCT	8	M age in yrs (SD), males (%), DM (%):[£] 57.7 (15.8), 44.4, NA.<br>KDIGO GFR G3a–G5/KRT (%): 100.0/0.0<br>Causes of CKD (%): NA.	Iron isomaltoside IV 1000 mg (single dose or two split doses of 500 mg) during 1st wk (233).	Iron sulphate PO 2 x 100 mg/d (118).	Changes in Hgb, SF, TSAT, Fe, and TIBC.<br>TEAE.<br>Quality of life.	None.
**REVOKE**<br>USA [21].	RCT	96	M age in yrs (SD), males (%), DM (%):[£] 65.5 (11.3), 76.5, 43.4.<br>KDIGO GFR G3a–G5/KRT (%): 100.0/0.0<br>Causes of CKD (%): NA.	Iron sucrose IV 200 mg/2 wks for 8 wks (67).	Iron sulphate PO 3 x 325 mg/d for 8 wks (69).	Change in Hgb.<br>Intensification of ESA therapy and/or need of blood transfusions.[§]<br>Change in eGFR and proteinuria.<br>Quality of life.	Scheduled adjustable ESA dosing (not all participants on ESA therapy).<br>Blood transfusions.
**CCF-II study**<br>Italy [22].	RCT	12	M age in yrs (SD), males (%), DM (%):[£] 50.4 (15.5), 56.8, NA.<br>KDIGO GFR G3a–G5/KRT (%): 100.0/0.0<br>Causes of CKD (%): GN/AID (30.0), DM (27.5), unknown/other (24.5), ADPKD (18.0).	Iron gluconate IV 125 mg/wk (37).	Liposomal iron PO 30/70 mg/d (69).	Changes in Hgb, SF and TSAT.<br>≥0.6 g/dL increase in Hgb.	Fixed ESA dosing (not all participants on ESA therapy).

**FIND-CKD**<br>NCT00994318<br>Global [23].	RCT	56	M age in yrs (SD), males (%), DM (%):£ 69.3 (13.4), 63.0, NA.<br>KDIGO GFR G3a–G5/KRT (%): 100.0/0.0<br>Causes of CKD (%): NA.	FCM IV 1000 + 500–1000 mg/4 wks for 48 wks (155).<br>FCM IV 2000 + 2000 mg/4 wks for 48 wks (154).	Iron sulphate PO 2 x 100 mg/d for 52 wks (317).	≥1 g/dL increase in Hgb.<br>Hgb ≥10.0 g/dL.<br>Initiation of ESA therapy and/or need of blood transfusions.§<br>Change in eGFR.<br>TEAE.<br>Quality of life.	Initiation of ESA if required after 8 wks post-randomization (not all participants on ESA therapy).<br>Blood transfusions.
**REPAIR-IDA**<br>NCT00981045<br>USA [24].	RCT<br>STR	14	M age in yrs (SD), males (%), DM (%):¶ 67.3 (13.0), 76.5, NA.<br>KDIGO GFR G3a–G5/KRT (%): 94.3/0.0<br>Causes of CKD (%): NA.	FCM IV 2 x 750 mg during 1st wk (1290).	Iron sucrose IV 5 x 200 mg during 2 wks (1294).	Changes in Hgb, SF and TSAT.<br>≥1 g/dL increase in Hgb.<br>TEAE.	Adjustable ESA dosing (not all participants on ESA therapy).
**Ferumoxytol authorization studies**<br>NCT00255424<br>USA [5, 25–27].	RCT	5	M age in yrs (SD), males (%), DM (%):¶ 65.6 (13.2), 46.2, NA.<br>KDIGO GFR G3a–G5/KRT (%): 98.3/0.0<br>Causes of CKD (%): NA.	Ferumoxytol IV 2 x 510 mg during 1st wk (454).	Iron fumarate PO 4 x 50 mg/d for 3 wks (153).	Changes in Hgb, SF, TSAT, Fe, TIBC, and CHr.<br>≥1 g/dL increase in Hgb.<br>TEAE.	Fixed ESA dosing (not all participants on ESA therapy).
**Qunibi et al., 2011**<br>USA [28].	RCT	8	M age in yrs (SD), males (%), DM (%):£ 66.1 (13.1), 32.6, NA.<br>KDIGO GFR G3a–G5/KRT (%): 100.0/0.0<br>Causes of CKD (%): NA.	FCM IV 1000 + 500 + 500 mg during 4 wks (152).	Iron sulphate PO 3 x 325 mg/d (103).	≥1 g/dL increase in Hb.<br>Changes in Hgb and SF.<br>Initiation of ESA therapy and/or need of blood transfusions.§<br>TEAE.	Fixed ESA dosing (not all participants on ESA therapy).
**McMahon et al., 2010**<br>Australia [29].	RCT	48	M age in yrs (SD), males (%), DM (%):£ 69.5 (10.0), 73.0, 47.0.<br>KDIGO GFR G3a–G5/KRT	Iron sucrose IV 100–200 mg/8 wks (52).	Iron sulphate PO 3 x 325 mg/d (48).	Change in Hgb.<br>Change in eGFR and time of	Initiation of ESA if required.

			(%): 100.0/0.0<br>Causes of CKD (%): NA.			commencing dialysis.<br>Initiation of ESA therapy.§<br>Quality of life.	
**US Venofer CT study 2005**<br>USA [30].	RCT<br>STR	8	M age in yrs (SD), males (%), DM (%):£ 63.1 (13.5), 32.3, NA.<br>KDIGO GFR G3a–G5/KRT (%): 100.0/0.0<br>Causes of CKD (%): NA	Iron sucrose IV 2 x 500 mg or 5 x 200 mg during 2 wks (95).	Iron sulphate PO 3 x 325 mg/d (93).	≥1 g/dL increase in Hb.<br>Changes in Hgb, SF, TSAT, and CHr.<br>TEAE.	Adjustable ESA dosing (not all participants on ESA therapy).
**Venofer CS study**<br>USA [31].	RCT	6	M age in yrs (SD), males (%), DM (%):£ 61.0 (14.2), 65.6, NA.<br>KDIGO GFR G3a–G5/KRT (%): 100.0/0.0<br>Causes of CKD (%): NA.	Iron sucrose IV 5 x 200 mg during 4 wks (48).	Iron sulphate PO 3 x 325 mg/d for 4 wks (48).	Changes in Hgb, SF and TSAT.<br>Hgb >11.0 g/dL.<br>TEAE.	Fixed ESA dosing.
**St James's University Hospital study**<br>UK [32].	RCT	24	M age in yrs (SD), males (%), DM (%):£ 59.3 (13.7), 55.6, 11.1.<br>KDIGO GFR G3a–G5/KRT (%): 100.0/0.0<br>Causes of CKD (%): unknown/other (33.4), GN/AID (22.2), TIN/HTN (20.0), ADPKD (13.3), DM (11.1).	Iron sucrose IV 300 mg/4 wks (22).	Iron sulphate PO 3 x 200 mg/d (23).	Changes in Hgb and SF.<br>Hgb ≥12.0 g/dL.<br>Intensification of ESA therapy.§	Fixed ESA dosing.

(**b**) The trials that did not provide numerical data for the meta-analysis.[&]

**Trial** Details	**Design**	**Follow-up** (in wks)	**Participants/population** Characteristics	**Interventions** (n)	**Comparators** (n)	**Outcomes**	**Co-interventions**
**Second Xiangya Hospital study** China [33].	RCT	8	M age in yrs (SD), males (%), DM (%):[$] 50.5 (1.6), 70.7, 19.0. KDIGO GFR G3a–G5/KRT (%): 0.0/100.0 Causes of CKD (%): TIN/HTN (58.6), DM (19.0), unknown/other (15.5), ADPKD (6.9).	Iron sucrose IV 2 x 25 mg/wk (19). Iron sucrose IV 100 mg/2 wks (20).	Non-iron (20). Healthy controls (20).	Changes in Hb, Hct, SF, TSAT, and oxidative stress parameters.	Adjustable ESA dosing.
**Mackay Memorial Hospital study** Taiwan [34].	RCT	24	M age in yrs (SD), males (%), DM (%):[$] 60.8 (13.6), 49.0, NA. KDIGO GFR G3a–G5/KRT (%): 0.0/100.0 Causes of CKD (%): NA.	Iron sucrose IV 100 mg/wk for 8 wks (51).	Iron chloride PO 100 mg/wk for 8 wks (47).	Changes in Hgb, SF and TSAT. Intensification of ESA therapy and/or need of blood transfusions.[§]	Scheduled adjustable ESA dosing. Blood transfusions.
**Sheashaa et al., 2005** Egypt [35].	RCT	24	M age in yrs (SD), males (%), DM (%):[$] 39.5 (13.6), 68.8, 0.0. KDIGO GFR G3a–G5/KRT (%): 0.0/100.0 Causes of CKD (%): TIN/HTN (41.7), GN/AID (27.1), unknown/other (22.9), ADPKD (8.3).	Iron saccharate IV 2 x 100 mg/wk for 8 wks followed by 100 mg/wk for 16 wks (22).	Iron gluconate IV 2 x 62.5 mg/wk for 8 wks followed by 62.5 mg/wk for 16 wks (26).	Changes in Hb, Hct, SF, TSAT, and Fe. TEAE.	None.
**Aggarwal et al., 2003** India [36].	RCT	12	M age in yrs (SD), males (%), DM (%):[$] 46.0 (17.0), 72.5, NA. KDIGO GFR G3a–G5/KRT (%): 0.0/100.0 Causes of CKD (%): NA.	Iron dextran IV 100 mg/2 wks (20).	Iron sulphate PO 3 x 200 mg/d (20).	Changes in Hb, SF, TSAT, Fe, TIBC, and BMIS.	Fixed ESA dosing.
**Nissenson et al., 1999**	RCT	7	M age in yrs (SD), males (%), DM (%):[$] 54.8 (17.3), 41.6, NA.	Iron gluconate IV 8 x 62.5 mg in	Iron sulphate PO 3 x 325 mg/d	Changes in Hb, Hct, SF, and TSAT.	Fixed ESA dosing.

USA [37].			KDIGO GFR G3a–G5/KRT (%): 0.0/100.0<br>Causes of CKD (%): NA.	8 HD sessions (47).<br>Iron gluconate IV 8 x 125 mg in 8 HD sessions (41).	or iron gluconate PO 3 x 650 mg/d (25).	TEAE.	
**Bronx-Lebanon Hospital Center study**<br>USA [38].	RCT	20	M age in yrs (SD), males (%), DM (%):$ 55.5 (11.0), 32.4, NA.<br>KDIGO GFR G3a–G5/KRT (%): 0.0/100.0<br>Causes of CKD (%): NA.	Iron dextran IV 100 mg/wk (18).	Iron sulphate PO 3 x 325 mg/d (19).	Changes in Hb, SF and TSAT.	Scheduled adjustable ESA dosing.
**Svára et al., 1996**<br>Czech Republic [39].	RCT	6	M age in yrs (SD), males (%), DM (%):$ 61.0 (6.0), 62.0, NA.<br>KDIGO GFR G3a–G5/KRT (%): 0.0/100.0<br>Causes of CKD (%): NA.	Iron saccharate IV 100 mg/wk (30).	Iron sulphate PO 3 x 325 mg/d (31).	Changes in Hgb, SF, TSAT, and Fe.	Adjustable ESA dosing.
**Royal London Hospital study**<br>UK [40].	RCT CO	52	M age in yrs (SD), males (%), DM (%):$ NA, NA, NA.<br>KDIGO GFR G3a–G5/KRT (%): 0.0/100.0<br>Causes of CKD (%): NA.	Iron dextran IV 100 mg/2 wks (15).	Iron sulphate PO 325 mg/d (15).	Changes in Hgb, MCH, MCHC, TSAT, Fe, TIBC, and BMIS.	None.
**Ottawa Hospital study**<br>NCT00318812<br>Canada [41].	RCT	24	M age in yrs (SD), males (%), DM (%):£ 71.0 (10.0), 62.5, 37.5.<br>KDIGO GFR G3a–G5/KRT (%): 100.0/0.0<br>Causes of CKD (%): DM (37.5), TIN/HTN (32.5), Unknown/other (30.0)	Iron sucrose IV 200 mg/4 wks (22).	HIP PO 3 x 11 mg/d (18)	Changes in Hb, SF, TSAT.	Scheduled adjustable ESA dosing.
**RMC study**<br>USA [42].	RCT	6	M age in yrs (SD), males (%), DM (%):£ 63.9 (14.1), 62.5, 37.5.<br>KDIGO GFR G3a–G5/KRT (%): 100.0/0.0<br>Causes of CKD (%): NA.	Iron gluconate IV 250 mg/wk for 4 wks (44).	Iron sulphate PO 2 x 325 mg/d (45).	Changes in Hgb, SF, TSAT, and CHr.<br>TEAE.<br>Quality of life.	None.

&Regardless of their inclusion in the meta-analysis, all the studies eligible were included in the systematic review.
$Study participants were ESKD patients, who had yet started KRT (only HD and PD modalities).
€Study participants were individuals with NKF/CKD KDIGO GFR categories G1–G2, patients with CKD KDIGO GFR categories G3a–G5, and patients treated with chronic dialysis.
£Study participants were individuals with CKD KDIGO GFR categories G3a–G5.
¥Study participants were individuals with CKD KDIGO GFR categories G3a–G5 and dialysis patients.
¶Study participants were individuals with NKF/CKD KDIGO GFR categories G1–G2 and patients with CKD KDIGO GFR categories G3a–G5 but not dialysis patients.
@Study participants were kidney transplant recipients in their early post-transplant period.
§Changes in anemia management included: initiation/intensification of ESA therapy, increase/change of iron supplement and need of blood transfusions.

Abbreviations: ADPKD, autosomal-dominant polycystic kidney disease; AID, autoimmune disease; Barts, St Bartholomew's Hospital; BMIS, bone marrow iron store; CCF-II, CKD Clinic of the University Federico II of Naples, Italy; CHr, reticulocyte hemoglobin content; CKD, chronic kidney disease; CNI, calcineurin inhibitor; CO, cross over trial design; COSMOS, Pharmacosmos A/S; CRP, C-reactive protein; DM, diabetes mellitus; DRIVE, Dialysis patients' Response to IV iron with Elevated ferritin; eGFR, estimated glomerular filtration rate; ESA, erythropoiesis-stimulating agent; ESKD, end-stage kidney disease; GN, glomerulonephritis; HD, hemodialysis; HF, heart failure; HTN, hypertension; KCH, King's College Hospital; KDIGO; Kidney Disease: Improving Global Outcomes; KfH, Kuratorium für Dialyse und Nierentransplantation e.V.; KRT, kidney replacement therapy; FACT, Ferumoxytol for Anemia of CKD Trial; FCM, ferric carboxymaltose; Fe, serum iron; FIND-CKD, Ferinject® assessment in patients with IroN Deficiency anaemia and non-dialysis-dependent Chronic Kidney Disease; Hgb, hemoglobin; Hct, hematocrit; HIP, Heme iron polypeptide; MCH, mean corpuscular hemoglobin; MCHC, mean corpuscular hemoglobin concentration; MRI, magnetic resonance imaging; NA, non-available; NKF, normal kidney function; PD, peritoneal dialysis; RAH, Royal Adelaide Hospital; Ret, reticulocyte count; REVOKE, Randomized trial to Evaluate intraVenous and Oral iron in chronic KidnEy disease; RMC, Richard L. Roudebush VA Medical Center; SF, serum ferritin; STR, stratified trial design; TIBC, total iron binding capacity; TIN, tubulo-interstitial nephritis; TP, treatment period; TSAT, transferrin saturation; TEAE, treatment-emergent adverse event; Venofer CS, Venofer Clinical Studies group; US Venofer CT, United States Iron Sucrose (Venofer) Clinical Trials group.

Table 3. Risk of bias in the eligible studies.

Trials	Random sequence generation	Allocation concealment	Blinding of participants and personnel	Blinding of outcome assessment	Incomplete outcome data	Selective reporting	Other bias
**FACT** [1]	L	L	U	U	U	L	U
**PIVOTAL** [2]	L	L	U	L	U	U	U
**COSMOS study 2015** [3]	L	L	U	U	L	L	U
**Princess Alexandra Hospital study** [4]	L	L	U	U	U	U	U
**Ferumoxytol authorization studies** (dialysis) [5, 6]	L	L	U	L	U	U	U
**DRIVE & DRIVE II** [7, 8]	L	L	U	U	U	U	U
**Beijing Chaoyang Hospital study** [9–11]	L	L	U	U	U	U	U
**US Venofer CT study 2006** [12]	L	L	U	U	U	L	U
**KfH Dialysis Centre study** [13]	L	L	U	U	U	U	U
**Barts study** [14]	L	L	U	U	U	U	U
**Winthrop University Hospital study** [15].	L	L	U	U	U	U	U
**KCH/RAH studies** [16–18]	L	L	U	U	U	U	U
**Luitpold Pharma study** [19]	L	L	U	U	U	L	U
**COSMOS study 2016** [20]	L	L	U	U	L	L	U
**REVOKE** [21]	L	L	U	U	U	U	U
**CCF-II study** [22]	L	L	U	U	U	L	U
**FIND-CKD** [23]	L	L	U	U	U	U	U
**REPAIR-IDA** [24]	L	L	U	L	L	U	U
**Ferumoxytol authorization studies** (CKD 3A-5) [5, 25–27]	L	L	U	L	U	U	U
**Qunibi et al., 2011** [28]	L	L	U	U	L	L	U

**McMahon et al., 2010** [29]	L	L	U	U	U	U	U
**US Venofer CT study 2005** [30]	L	L	U	U	U	U	U
**Venofer CS study** [31]	L	L	U	U	U	U	U
**St James's University Hospital study** [32].	L	L	U	U	L	U	U
**Second Xiangya Hospital study** [33]	L	U	U	U	U	U	U
**Mackay Memorial Hospital study** [34]	L	L	U	U	U	U	U
**Sheashaa et al., 2005** [35]	L	L	U	U	U	U	U
**Aggarwal et al., 2003** [36]	L	L	U	U	U	U	U
**Nissenson et al., 1999** [37]	L	L	U	U	U	U	U
**Bronx-Lebanon Hospital Center study** [38]	L	L	U	U	U	U	U
**Svára et al., 1996** [39]	L	U	U	U	U	U	U
**Royal London Hospital study** [40]	L	L	L	U	L	U	U
**Ottawa Hospital study** [41]	L	L	L	U	L	L	U
**RMC study** [42]	L	L	U	U	U	U	U

The judgement for each of risk of bias domain is presented as (L), (U) or (H) to indicate low, unclear, or high risk of bias, respectively.

a

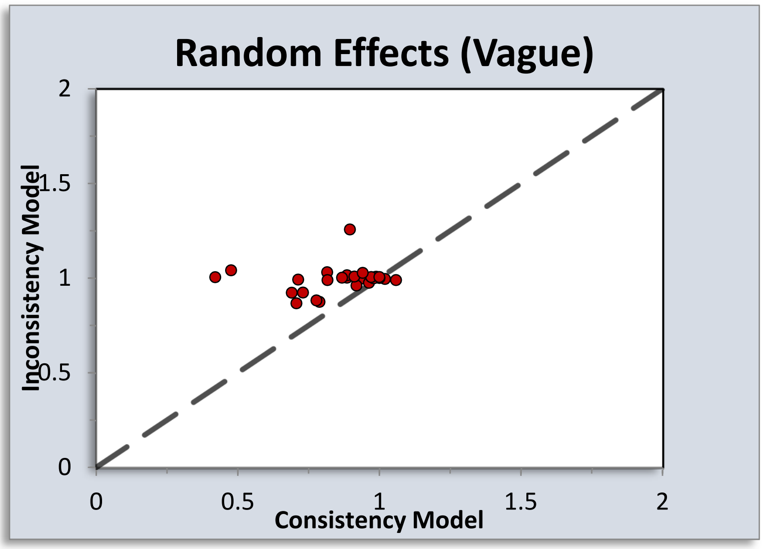

b

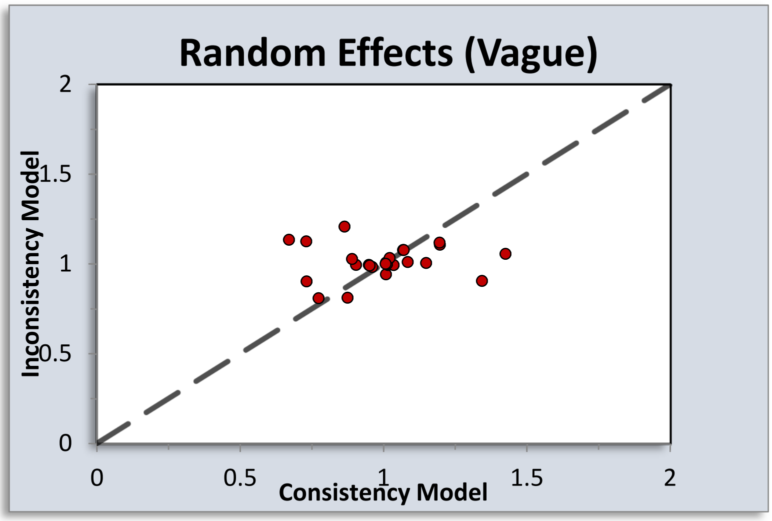

Figure S1. Inconsistency plot on random effects for the SUCRA assessable iron supplements values in the subgroups of (**a**) patients in the KDIGO GFR categories 3A to 5, and (**b**) dialysis patients. GFR, Glomerular filtration rate; KDIGO, Kidney Disease—Improving Global Outcomes.

REFERENCES

1. Macdougall, I.C.; Strauss, W.E.; Dahl, N.V.; Bernard, K.; Li, Z. Ferumoxytol for iron deficiency anemia in patients undergoing hemodialysis: The FACT randomized controlled trial. *Clin. Nephrol.* **2019**, *91*, 237–245. https://doi.org/10.5414/CN109512
2. Macdougall, I.C.; White, C.; Anker, S.D.; Bhandari, S.; Farrington, K.; Kalra, P.A.; McMurray, J.J.V.; Murray, H.; Tomson, C.R.V.; Wheeler, D.C.; et al. Intravenous iron in patients undergoing maintenance hemodialysis. *N Engl J Med* 2019; 380: 447–458. https://doi.org.10.1056/NEJMoa1810742
3. Bhandari, S.; Kalra, P.A.; Kothari, J.; Ambühl, P.M.; Christensen, J.H.; Essaian, A.M.; Thomsen, L.L.; Macdougall, I.C.; Coyne, D.W. A randomized, open-label trial of iron isomaltoside 1000 (Monofer®) compared with iron sucrose (Venofer®) as maintenance therapy in haemodialysis patients. *Nephrol. Dial. Transplant.* **2015**, *30*, 1577–1589. https://doi.org/10.1093/ndt/gfv096
4. Mudge, D.W.; Tan, K.S.; Miles, R.; Johnson, D.W.; Badve, S.V.; Campbell, S.B.; Isbel, N.M.; van Eps, C.L.; Hawley, C.M. A randomized controlled trial of intravenous or oral iron for posttransplant anemia in kidney transplantation. *Transplantation* **2012**, *93*, 822–826. https://doi.org/10.1097/TP.0b013e318248375a
5. Lu, M.; Cohen, M.H.; Rieves, D.; Pazdur, R. FDA report: Ferumoxytol for intravenous iron therapy in adult patients with chronic kidney disease. *Am. J. Hematol.* **2010**, *85*, 315–319. https://doi.org/10.1002/ajh.21656
6. Provenzano, R.; Schiller, B.; Rao, M.; Coyne, D.; Brenner, L.; Pereira, B.J. Ferumoxytol as an intravenous iron replacement therapy in hemodialysis patients. *Clin. J. Am. Soc. Nephrol.* **2009**, *4*, 386–393. https://doi.org/10.2215/CJN.02840608
7. Coyne, D.W.; Kapoian, T.; Suki, W.; Singh, A.K.; Moran, J.E.; Dahl, N.V.; Rizkala, A.R. Ferric gluconate is highly efficacious in anemic hemodialysis patients with high serum ferritin and low transferrin saturation: Results of the Dialysis Patients' Response to IV Iron with Elevated Ferritin (DRIVE) Study. *J Am Soc Nephrol* **2007**, *18*, 975–984. https://doi.org/10.1681/ASN.2006091034
8. Kapoian, T.; O'Mara, N.B.; Singh, A.K.; Moran, J.; Rizkala, A.R.; Geronemus, R.; Kopelman, R.C.; Dahl, N.V.; Coyne, D.W. Ferric gluconate reduces epoetin requirements in hemodialysis patients with elevated ferritin. *J Am Soc Nephrol* **2008**, *19*, 372–379. https://doi.org/10.1681/ASN.2007050606
9. Li, H.; Wang, S.X. [Intravenous iron sucrose in maintenance dialysis patients with renal anemia: a clinical study]. *Zhonghua Yi Xue Za Zhi* **2009**, *89*, 457–462. https://doi.org/10.3760/cma.j.issn.0376-2491.2009.07.006
10. Li, H.; Wang, S.X. Intravenous iron sucrose in Chinese hemodialysis patients with renal anemia. *Blood Purif.* **2008**, *26*, 151–156. https://doi.org/10.1159/000113529
11. Li, H.; Wang, S.X. Intravenous iron sucrose in peritoneal dialysis patients with renal anemia. *Perit. Dial. Int.* **2008**, *28*, 149–154.
12. Singh, H.; Reed, J.; Noble, S.; Cangiano, J.L.; Van Wyck, D.B. Effect of intravenous iron sucrose in peritoneal dialysis patients who receive erythropoiesis-stimulating agents for anemia: A randomized, controlled trial. *Clin. J. Am. Soc. Nephrol.* **2006**, *1*, 475–482. https://doi.org/10.2215/CJN.01541005
13. Kosch, M.; Bahner, U.; Bettger, H.; Matzkies, F.; Teschner, M.; Schaefer, R.M. A randomized, controlled parallel-group trial on efficacy and safety of iron sucrose (Venofer) vs iron gluconate (Ferrlecit) in haemodialysis patients treated with rHuEpo. *Nephrol. Dial. Transplant.* **2001**, *16*, 1239–1244. https://doi.org/10.1093/ndt/16.6.1239
14. Macdougall, I.C.; Tucker, B.; Thompson, J.; Tomson, C.R.; Baker, L.R.; Raine, A.E. A randomized controlled study of iron supplementation in patients treated with erythropoietin. *Kidney Int.* **1996**; *50*, 1694–1699. https://doi.org/10.1038/ki.1996.487

15. Fishbane, S.; Frei, G.L.; Maesaka, J. Reduction in recombinant human erythropoietin doses by the use of chronic intravenous iron supplementation. *Am. J. Kidney. Dis.* **1995**, *26*, 41–46. https://doi.org/10.1016/0272-6386(95)90151-5
16. Strauss, W.E.; Dahl, N.V.; Li, Z.; Lau, G.; Allen, L.F. Ferumoxytol versus iron sucrose treatment: A post-hoc analysis of randomized controlled trials in patients with varying renal function and iron deficiency anemia. *BMC Hematol.* **2016**, *16*, 20. https://doi.org/10.1186/s12878-016-0060-x
17. Macdougall, I.C.; Strauss, W.E.; McLaughlin, J.; Li, Z.; Dellanna, F.; Hertel, J. A randomized comparison of ferumoxytol and iron sucrose for treating iron deficiency anemia in patients with CKD. *Clin. J. Am. Soc. Nephrol.* **2014**, *9*, 705–712. https://doi.org/10.2215/CJN.05320513
18. Hetzel, D.; Strauss, W.; Bernard, K.; Li, Z.; Urboniene, A.; Allen, L.F. A Phase III, randomized, open-label trial of ferumoxytol compared with iron sucrose for the treatment of iron deficiency anemia in patients with a history of unsatisfactory oral iron therapy. *Am. J. Hematol.* **2014**, *89*, 646–650. https://doi.org/10.1002/ajh.23712
19. Charytan, C.; Bernardo, M.V.; Koch, T.A.; Butcher, A.; Morris, D.; Bregman, D.B. Intravenous ferric carboxymaltose versus standard medical care in the treatment of iron deficiency anemia in patients with chronic kidney disease: A randomized, active-controlled, multi-center study. *Nephrol. Dial. Transplant.* **2013**, *28*, 953–964. https://doi.org/10.1093/ndt/gfs528
20. Kalra, P.A.; Bhandari, S.; Saxena, S.; Agarwal, D.; Wirtz, G.; Kletzmayr, J.; Thomsen, L.L.; Coyne, D.W. A randomized trial of iron isomaltoside 1000 versus oral iron in non-dialysis-dependent chronic kidney disease patients with anaemia. *Nephrol. Dial. Transplant.* **2016**, *31*, 646–655. https://doi.org/10.1093/ndt/gfv293
21. Agarwal, R.; Kusek, J.W.; Pappas, M.K. A randomized trial of intravenous and oral iron in chronic kidney disease. *Kidney Int.* **2015**, *88*, 905–914. https://doi.org/10.1038/ki.2015.163
22. Pisani, A.; Riccio, E.; Sabbatini, M.; Andreucci, M.; Del Rio, A.; Visciano, B. Effect of oral liposomal iron versus intravenous iron for treatment of iron deficiency anaemia in CKD patients: A randomized trial. *Nephrol. Dial. Transplant.* **2015**, *30*, 645–652. https://doi.org/10.1093/ndt/gfu357
23. Macdougall, I.C.; Bock, A.H.; Carrera, F.; Eckardt, K.U.; Gaillard, C.; Van Wyck, D.; Roubert, B.; Nolen, J.G.; Roger, S.D. FIND-CKD: A randomized trial of intravenous ferric carboxymaltose versus oral iron in patients with chronic kidney disease and iron deficiency anaemia. *Nephrol. Dial. Transplant.* **2014**, *29*, 2075–2084. https://doi.org/10.1093/ndt/gfu201
24. Onken, J.E.; Bregman, D.B.; Harrington; Morris, D.; Buerkert, J.; Hamerski, D.; Iftikhar, H.; Mangoo-Karim, R.; Martin, E.R.; Martinez, C.O.; et al. Ferric carboxymaltose in patients with iron-deficiency anemia and impaired renal function: the REPAIR-IDA trial. *Nephrol. Dial. Transplant.* **2014**, *29*, 833–842. https://doi.org/10.1093/ndt/gft251
25. Fishbane, S.; Bolton, W.K.; Winkelmayer, W.C.; Strauss, W.; Li, Z.; Pereira, B.J. Factors affecting response and tolerability to ferumoxytol in nondialysis chronic kidney disease patients. *Clin. Nephrol.* **2012**, *78*, 181–188. https://doi.org/10.5414/cn107397
26. Bolton, W.K.; Fishbane, S.; Li, J.; Milich, L.; Brenner, R. 29: Increases in hemoglobin and the effect of ESA use in CKD patients treated with IV ferumoxytol [abstract]. *Am. J. Kidney Dis.* **2009**, *53*, B29. https://doi.org/10.1053/j.ajkd.2009.01.050
27. Spinowitz, B.S.; Kausz, A.T.; Baptista, J.; Noble, S.D.; Sothinathan, R.; Bernardo, M.V.; Brenner, L.; Pereira, B.J. Ferumoxytol for treating iron deficiency anemia in CKD. *J. Am. Soc. Nephrol.* **2008**, *19*, 1599–1605. https://doi.org/10.1681/ASN.2007101156
28. Qunibi, W.Y.; Martinez, C.; Smith, M.; Benjamin, J.; Mangione, A.; Roger, S.D. A randomized controlled trial comparing intravenous ferric carboxymaltose with oral iron for treatment of iron deficiency anaemia of non-dialysis-dependent chronic kidney disease patients. *Nephrol. Dial. Transplant.* **2011**, *26*, 1599–1607. https://doi.org/10.1093/ndt/gfq613
29. McMahon, L.P.; Kent, A.B.; Kerr, P.G.; Healy, H.; Irish, A.B.; Cooper, B.; Kark, A.; Roger, S.D. Maintenance of elevated versus physiological iron indices in non-anaemic patients with

chronic kidney disease: a randomized controlled trial. *Nephrol. Dial. Transplant.* **2010**, *25*, 920–926. https://doi.org/10.1093/ndt/gfp584
30. Van Wyck, D.B.; Roppolo, M.; Martinez, C.O.; Mazey, R.M.; McMurray, S. A randomized, controlled trial comparing IV iron sucrose to oral iron in anemic patients with nondialysis-dependent CKD. *Kidney Int.* **2005**, *68*, 2846–2856. https://doi.org/10.1111/j.1523-1755.2005.00758.x
31. Charytan, C.; Qunibi, W.; Bailie, G.R. Comparison of intravenous iron sucrose to oral iron in the treatment of anemic patients with chronic kidney disease not on dialysis. *Nephron. Clin. Pract.* **2005**, *100*, c55–c62. https://doi.org/10.1159/000085049
32. Stoves, J.; Inglis, H.; Newstead, C.G. A randomized study of oral vs intravenous iron supplementation in patients with progressive renal insufficiency treated with erythropoietin. *Nephrol. Dial. Transplant.* **2001**, *16*, 967–974. https://doi.org/10.1093/ndt/16.5.967
33. Yin, L.; Chen, X.; Chen, J.; Cheng, M.; Peng, Y.; Yang L. [Multi-frequency low-dose intravenous iron on oxidative stress in maintenance hemodialysis patients]. *Zhong Nan Da Xue Xue Bao Yi Xue Ban* **2012**, 37, 844–848. https://doi.org/10.3969/j.issn.1672-7347.2012.08.015
34. Wu, C.J.; Lin, H.C.; Lee, K.F.; Chuang, C.K.; Chen, Y.C.; Chen, H.H. Comparison of parenteral iron sucrose and ferric chloride during erythropoietin therapy of haemodialysis patients. *Nephrology (Carlton)* **2010**, *15*, 42–47. https://doi.org/10.1111/j.1440-1797.2009.01160.x
35. Sheashaa, H.; El-Husseini, A; Sabry, A; Hassan, N.; Salem, A.; Khalil, A.; El-Agroudy, A.; Sobh, M. Parenteral iron therapy in treatment of anemia in end-stage renal disease patients: a comparative study between iron saccharate and gluconate. *Nephron. Clin. Pract.* **2005**, *99*, c97–c101. https://doi.org/10.1159/000083766
36. Aggarwal, H.K.; Nand, N.; Singh, S.; Singh, M.; Hemant, A.; Kaushik, G. Comparison of oral versus intravenous iron therapy in predialysis patients of chronic renal failure receiving recombinant human erythropoietin. *J. Assoc. Physicians India* **2003**, *51*, 170–174.
37. Nissenson, A.R.; Lindsay, R.M.; Swan, S.; Seligman, P.; Strobos, J. Sodium ferric gluconate complex in sucrose is safe and effective in hemodialysis patients: North American Clinical Trial. *Am. J. Kidney Dis.* **1999**, *33*, 471–482. https://doi.org/10.1016/S0272-6386(99)70184-8
38. Kotaki, M.; Uday, K.; Henriquez, M.; Blum, S.; Dave, M. Maintenance therapy with intravenous iron in hemodialysis patients receiving erythropoietin. *Clin. Nephrol.* **1997**, *48*, 63–64.
39. Svára, F.; Sulková, S.; Kvasnićka, J.; Polakovic, V. [Iron supplementation during erythropoietin therapy in patients on hemodialysis]. *Vnitr. Lek* **1996**, *42*, 849–852.
40. Strickland, I.D.; Chaput de Saintonge, D.M.; Boulton, F.E.; Francis, B., Roubikova, J.; Waters, J.I. The therapeutic equivalence of oral and intravenous iron in renal dialysis patients. *Clin. Nephrol.* **1977**, *7*, 55–57.
41. Nagaraju, S.P.; Cohn, A.; Akbari, A.; Davis, J.L.; Zimmerman, D.L. Heme iron polypeptide for the treatment of iron deficiency anemia in non-dialysis chronic kidney disease patients: a randomized controlled trial. *BMC Nephrol.* **2013**, *14*, 64. https://doi.org/10.1186/1471-2369-14-64
42. Agarwal, R.; Rizkala, A.R.; Bastani, B.; Kaskas, M.O.; Leehey, D.J.; Besarab, A. A randomized controlled trial of oral versus intravenous iron in chronic kidney disease. *Am. J. Nephrol.* **2006**, *26*, 445–454. https://doi.org/10.1159/000096174

Panel S1. Search strategy formulae and search results.

Formulae	Records
**PubMed**	
"Kidney Diseases"[Mesh]	506868
"kidney disease"[Text Word]	74052
("kidney"[Text Word]) OR "renal"[Text Word]	1061436
"Kidney Function Tests"[Mesh]	78470
"Glomerular Filtration Rate"[Mesh]	43504
"creatinine"[Text Word]	132085
(("proteinuria"[Text Word]) OR "microalbuminuria"[Text Word]) OR "macroalbuminuria"[Text Word]	55995
"Renal Insufficiency, Chronic"[Mesh]	113070
"Kidney Failure, Chronic"[Mesh]	92576
"Uremia"[Mesh]	24489
("uremia"[Text Word]) OR "uraemia"[Text Word]	23177
"Renal Replacement Therapy"[Mesh]	208388
"Renal Dialysis"[Mesh]	112509
"Hemodiafiltration"[Mesh]	2317
"Hemodialysis, Home"[Mesh]	1915
"Peritoneal Dialysis"[Mesh]	25989
"Peritoneal Dialysis, Continuous Ambulatory"[Mesh]	9911
(("dialysis"[Text Word]) OR "hemodialysis"[Text Word]) OR "haemodialysis"[Text Word]	181544
(((("ultrafiltration"[Text Word]) OR "hemofiltration"[Text Word]) OR "haemofiltration"[Text Word]) OR "hemodiafiltration"[Text Word]) OR "haemodiafiltration"[Text Word]	28304
"Kidney Transplantation"[Mesh]	94397
(("transplant"[Text Word]) OR "transplantation"[Text Word]) OR "transplanted"[Text Word]	733124
"Anemia"[Mesh]	159644
((("anemia"[Text Word]) OR "anaemia"[Text Word]) OR "anemic"[Text Word]) OR "anaemic"[Text Word]	195245
((((("Iron Compounds"[Mesh]) OR "Ferric Oxide, Saccharated"[Mesh]) OR "Ferrosoferric Oxide"[Mesh]) OR "Iron Carbonyl Compounds"[Mesh]) OR "Iron, Dietary"[Mesh]) OR "Iron-Dextran Complex"[Mesh]	69751
((("iron"[Text Word]) OR "intravenous"[Text Word]) OR "oral"[Text Word]) OR "per os"[Text Word]	1230305
("Erythropoietin"[Mesh]) OR "Epoetin Alfa"[Mesh]	23404
(("epoetin"[Text Word]) OR "darbepoetin"[Text Word]) OR "erythropoietin"[Text Word]	31805
"erythropoiesis"[Text Word]	20952
(("Hemoglobins"[Mesh]) OR "Transferrin"[Mesh]) OR "Ferritins"[Mesh]	155934
((("hemoglobin"[Text Word]) OR "haemoglobin"[Text Word]) OR "transferrin"[Text Word]) OR "ferritin"[Text Word]	228024
(("Clinical Trials as Topic"[Majr:NoExp]) OR "Controlled Clinical Trials as Topic"[Majr:NoExp]) OR "Randomized Controlled Trials as Topic"[Majr:NoExp]	50035
"trial"[Text Word]	1174999
"Random Allocation"[Majr:NoExp]	721

((("randomized"[Text Word]) OR "random"[Text Word]) OR "randomization"[Text Word]) OR "randomly"[Text Word]	683315
("Double-Blind Method"[Majr:NoExp]) OR "Single-Blind Method"[Majr:NoExp]	848
(("double blind"[Text Word]) OR "single blind"[Text Word]) OR "open label"[Text Word]	261911
"Placebos"[Majr:NoExp]	3004
"placebo"[Text Word]	212666
**Ovid**	
exp Kidney Diseases/<br>OvidMEDLINE(R) ALL 1946 to March 31, 2020	506892
(kidney adj disease).tw.<br>Journals@Ovid<br>Subscribed Ovid journals by Sacyl<br>Ovid MEDLINE(R) ALL 1946 to March 31, 2020	178531
(kidney or renal).tw.<br>Journals@Ovid<br>Subscribed Ovid journals by Sacyl<br>Ovid MEDLINE(R) ALL 1946 to March 31, 2020	1829983
exp Kidney Function Tests/<br>OvidMEDLINE(R) ALL 1946 to March 31, 2020	78450
exp Glomerular Filtration Rate/<br>OvidMEDLINE(R) ALL 1946 to March 31, 2020	43485
creatinine.tw.<br>Journals@Ovid<br>Subscribed Ovid journals by Sacyl<br>Ovid MEDLINE(R) ALL 1946 to March 31, 2020	370923
(proteinur$ or microalbuminur$ or macroalbuminur$).tw.<br>Journals@Ovid<br>Subscribed Ovid journals by Sacyl<br>Ovid MEDLINE(R) ALL 1946 to March 31, 2020	125055
exp Renal Insufficiency, Chronic/<br>OvidMEDLINE(R) ALL 1946 to March 31, 2020	113038
exp Kidney Failure, Chronic/<br>OvidMEDLINE(R) ALL 1946 to March 31, 2020	92568
exp Uremia/<br>OvidMEDLINE(R) ALL 1946 to March 31, 2020	24485
ur?emi$.tw.<br>Journals@Ovid<br>Subscribed Ovid journals by Sacyl<br>Ovid MEDLINE(R) ALL 1946 to March 31, 2020	64431
exp Renal Replacement Therapy/<br>OvidMEDLINE(R) ALL 1946 to March 31, 2020	208367
exp Renal Dialysis/<br>OvidMEDLINE(R) ALL 1946 to March 31, 2020	112496
exp Hemodiafiltration/<br>OvidMEDLINE(R) ALL 1946 to March 31, 2020	2315
exp Hemodialysis, Home/<br>OvidMEDLINE(R) ALL 1946 to March 31, 2020	1915
exp Peritoneal Dialysis/<br>OvidMEDLINE(R) ALL 1946 to March 31, 2020	25990
exp Peritoneal Dialysis, Continuous Ambulatory/	9911

OvidMEDLINE(R) ALL 1946 to March 31, 2020	
(dialysis or h?emodialysis).tw.<br>Journals@Ovid<br>Subscribed Ovid journals by Sacyl<br>Ovid MEDLINE(R) ALL 1946 to March 31, 2020	352454
(ultrafiltration or h?emofiltration or h?emodiafiltration).tw.<br>Journals@Ovid<br>Subscribed Ovid journals by Sacyl<br>Ovid MEDLINE(R) ALL 1946 to March 31, 2020	52474
exp Kidney Transplantation/<br>OvidMEDLINE(R) ALL 1946 to March 31, 2020	94387
transplant$.tw.<br>Journals@Ovid<br>Subscribed Ovid journals by Sacyl<br>Ovid MEDLINE(R) ALL 1946 to March 31, 2020	1115973
exp Anemia/<br>OvidMEDLINE(R) ALL 1946 to March 31, 2020	159650
an?emi$.tw.<br>Journals@Ovid<br>Subscribed Ovid journals by Sacyl<br>Ovid MEDLINE(R) ALL 1946 to March 31, 2020	369682
exp Iron Compounds/ or exp Ferric Oxide, Saccharated/ or exp Ferrosoferric Oxide/ or exp Iron Carbonyl Compounds/ or exp Iron, Dietary/ or exp Iron-Dextran Complex/<br>OvidMEDLINE(R) ALL 1946 to March 31, 2020	69741
(iron or intraven$ or oral$).tw. or (per adj os).tw.<br>Journals@Ovid<br>Subscribed Ovid journals by Sacyl<br>Ovid MEDLINE(R) ALL 1946 to March 31, 2020	2507011
exp Erythropoietin/ or exp Epoetin Alfa/<br>Ovid MEDLINE(R) ALL 1946 to March 31, 2020	23407
(epoetin or darbepoetin or erythrop$).tw.<br>Journals@Ovid<br>Subscribed Ovid journals by Sacyl<br>Ovid MEDLINE(R) ALL 1946 to March 31, 2020	96413
exp Hemoglobins/ or exp Transferrin/ or exp Ferritins/<br>Ovid MEDLINE(R) ALL 1946 to March 31, 2020	155945
(h?emoglobin or transferrin or ferritin).tw.<br>Journals@Ovid<br>Subscribed Ovid journals by Sacyl<br>Ovid MEDLINE(R) ALL 1946 to March 31, 2020	212854
*Clinical Trials as Topic/ or *Controlled Clinical Trials as Topic/ or *Randomized Controlled Trials as Topic/<br>OvidMEDLINE(R) ALL 1946 to March 31, 2020	50035
trial$.tw.<br>Journals@Ovid<br>Subscribed Ovid journals by Sacyl<br>Ovid MEDLINE(R) ALL 1946 to March 31, 2020	2748674
*Random Allocation/<br>OvidMEDLINE(R) ALL 1946 to March 31, 2020	721
random$.tw.<br>Journals@Ovid	3076136

Subscribed Ovid journals by Sacyl<br>Ovid MEDLINE(R) ALL 1946 to March 31, 2020	
*Double-Blind Method/ or *Single-Blind Method/<br>OvidMEDLINE(R) ALL 1946 to March 31, 2020	848
(doubl$ adj blind$).tw. or (singl$ adj blind$).tw. or open label.tw.<br>Journals@Ovid<br>Subscribed Ovid journals by Sacyl<br>Ovid MEDLINE(R) ALL 1946 to March 31, 2020	526312
*Placebos/<br>Ovid MEDLINE(R) ALL 1946 to March 31, 2020	3004
placebo$.tw.<br>Journals@Ovid<br>Subscribed Ovid journals by Sacyl<br>Ovid MEDLINE(R) ALL 1946 to March 31, 2020	611767
**Elsevier's Scopus**	
KEY ("kidney disease") AND NOT INDEX (medline)	27827
TITLE-ABS-KEY ("kidney disease") AND NOT INDEX (medline)	37017
(TITLE-ABS-KEY (kidney) OR TITLE-ABS-KEY (renal)) AND NOT INDEX (medline)	293468
KEY ("kidney function") AND NOT INDEX (medline)	21432
KEY ("glomerulus filtration rate") AND NOT INDEX (medline)	10104
TITLE-ABS-KEY (creatinine) AND NOT INDEX (medline)	49157
(TITLE-ABS-KEY (proteinur*) OR TITLE-ABS-KEY (microalbuminur*) OR TITLE-ABS-KEY (macroalbuminur*)) AND NOT INDEX (medline)	18343
KEY ("end stage renal disease") AND NOT INDEX (medline)	4764
KEY ("chronic kidney failure") AND NOT INDEX (medline)	11591
KEY (uremia) AND NOT INDEX (medline)	4277
TITLE-ABS-KEY (ur?emi*) AND NOT INDEX (medline)	1268
KEY ("renal replacement therapy") AND NOT INDEX (medline)	6158
KEY (hemodialysis) AND NOT INDEX (medline)	24735
KEY (hemodiafiltration) AND NOT INDEX (medline)	754
KEY ("home dialysis") AND NOT INDEX (medline)	453
KEY ("peritoneal dialysis") AND NOT INDEX (medline)	6549
KEY ("continuous ambulatory peritoneal dialysis") AND NOT INDEX (medline)	1790
(TITLE-ABS-KEY (dialysis) OR TITLE-ABS-KEY (h?emodialysis)) AND NOT INDEX (medline)	45088
(TITLE-ABS-KEY (ultrafiltration) OR TITLE-ABS-KEY (h?emofiltration) OR TITLE-ABS-KEY (h?emodiafiltration)) AND NOT INDEX (medline)	25810
KEY ("kidney transplantation") AND NOT INDEX (medline)	16833
TITLE-ABS-KEY (transplant*) AND NOT INDEX (medline)	171415
KEY (anemia) AND NOT INDEX (medline)	52920
TITLE-ABS-KEY (an?emi*) AND NOT INDEX (medline)	14058
(KEY ("iron compounds") OR KEY ("Ferric Saccharate") OR KEY ("Iron Oxide (Saccharated)") OR KEY ("Ferriferrous Oxide") OR KEY (magnetite) OR KEY ("Iron Carbonyl Compounds") OR KEY ("Dietary Iron") OR KEY ("Dextran-Iron Complex")) AND NOT INDEX (medline)	83549
(TITLE-ABS-KEY (iron) OR TITLE-ABS-KEY (intraven*) OR TITLE-ABS-KEY (oral*) OR TITLE-ABS-KEY ("per os")) AND NOT INDEX (medline)	1101677
(KEY (erythropoietin) OR KEY (binocrit) OR KEY (eprex)) AND NOT INDEX (	9790

medline)	
(TITLE-ABS-KEY (epoetin) OR TITLE-ABS-KEY (darbepoetin) OR TITLE-ABS-KEY (erythrop*)) AND NOT INDEX (medline)	18312
(KEY (hemoglobins) OR KEY (transferrin) OR KEY (ferritins)) AND NOT INDEX (medline)	67977
(TITLE-ABS-KEY (h?emoglobin) OR TITLE-ABS-KEY (transferrin) OR TITLE-ABS-KEY (ferritins)) AND NOT INDEX (medline)	20354
(KEY ("clinical trial (topic)") OR KEY ("controlled clinical trial (topic)") OR KEY ("randomized controlled trial (topic)") AND NOT INDEX (medline)	67602
TITLE-ABS-KEY (trial) AND NOT INDEX (medline)	653893
KEY (randomization) AND NOT INDEX (medline)	13893
TITLE-ABS-KEY (random*) AND NOT INDEX (medline)	1264945
(KEY ("double blind procedure") OR KEY ("single blind procedure")) AND NOT INDEX (medline)	28814
(TITLE-ABS-KEY ("doubl* blind*") OR TITLE-ABS-KEY ("singl* blind*") OR TITLE-ABS-KEY ("open label")) AND NOT INDEX (medline)	57938
KEY (placebo) AND NOT INDEX (medline)	69781
TITLE-ABS-KEY (placebo) AND NOT INDEX (medline)	94575
**Web of Science**	
TOPIC: ("kidney disease")<br>Web of Science Core Collection<br>Current Contents Connect<br>Derwent Innovations Index<br>KCI-Korean Journal Database<br>Medline<br>Russian Science Citation Index<br>SciELO Citation Index	132574
TOPIC: (kidney) OR TOPIC: (renal)<br>Web of Science Core Collection<br>Current Contents Connect<br>Derwent Innovations Index<br>KCI-Korean Journal Database<br>Medline<br>Russian Science Citation Index<br>SciELO Citation Index	1727887
TOPIC: (creatinine)<br>Web of Science Core Collection<br>Current Contents Connect<br>Derwent Innovations Index<br>KCI-Korean Journal Database<br>Medline<br>Russian Science Citation Index<br>SciELO Citation Index	196518
TOPIC: (proteinur*) OR TOPIC: (microalbuminur*) OR TOPIC: (macroalbuminur*)<br>Web of Science Core Collection<br>Current Contents Connect<br>Derwent Innovations Index<br>KCI-Korean Journal Database<br>Medline<br>Russian Science Citation Index<br>SciELO Citation Index	84153

TOPIC: (ur?emi*)<br>Web of Science Core Collection<br>Current Contents Connect<br>Derwent Innovations Index<br>KCI-Korean Journal Database<br>Medline<br>Russian Science Citation Index<br>SciELO Citation Index	10084
TOPIC: (dialysis) OR TOPIC: (h?emodialysis)<br>Web of Science Core Collection<br>Current Contents Connect<br>Derwent Innovations Index<br>KCI-Korean Journal Database<br>Medline<br>Russian Science Citation Index<br>SciELO Citation Index	283900
TOPIC: (ultrafiltration) OR TOPIC: (h?emofiltration) OR TOPIC: (h?emodiafiltration)<br>Web of Science Core Collection<br>Current Contents Connect<br>Derwent Innovations Index<br>KCI-Korean Journal Database<br>Medline<br>Russian Science Citation Index<br>SciELO Citation Index	62729
TOPIC: (transplant*)<br>Web of Science Core Collection<br>Current Contents Connect<br>Derwent Innovations Index<br>KCI-Korean Journal Database<br>Medline<br>Russian Science Citation Index<br>SciELO Citation Index	1245092
TOPIC: (an?emi*)<br>Web of Science Core Collection<br>Current Contents Connect<br>Derwent Innovations Index<br>KCI-Korean Journal Database<br>Medline<br>Russian Science Citation Index<br>SciELO Citation Index	75645
TOPIC: (iron) OR TOPIC: (intraven*) OR TOPIC: (oral*) OR TOPIC: ("per os")<br>Web of Science Core Collection<br>Current Contents Connect<br>Derwent Innovations Index<br>KCI-Korean Journal Database<br>Medline<br>Russian Science Citation Index<br>SciELO Citation Index	2211263
TOPIC: (epoetin) OR TOPIC: (darbepoetin) OR TOPIC: (erythrop*)<br>Web of Science Core Collection<br>Current Contents Connect	89350

Derwent Innovations Index<br>KCI-Korean Journal Database<br>Medline<br>Russian Science Citation Index<br>SciELO Citation Index	
TOPIC: (h?emoglobin) OR TOPIC: (transferrin) OR TOPIC: (ferritin)<br>Web of Science Core Collection<br>Current Contents Connect<br>Derwent Innovations Index<br>KCI-Korean Journal Database<br>Medline<br>Russian Science Citation Index<br>SciELO Citation Index	163982
TOPIC: (trial)<br>Web of Science Core Collection<br>Current Contents Connect<br>Derwent Innovations Index<br>KCI-Korean Journal Database<br>Medline<br>Russian Science Citation Index<br>SciELO Citation Index	2134860
TOPIC: (random*)<br>Web of Science Core Collection<br>Current Contents Connect<br>Derwent Innovations Index<br>KCI-Korean Journal Database<br>Medline<br>Russian Science Citation Index<br>SciELO Citation Index	2545575
TOPIC: ("doubl* blind*") OR TOPIC: ("singl* blind*") OR TOPIC: ("open label")<br>Web of Science Core Collection<br>Current Contents Connect<br>Derwent Innovations Index<br>KCI-Korean Journal Database<br>Medline<br>Russian Science Citation Index<br>SciELO Citation Index	454973
TOPIC: (placebo)<br>Web of Science Core Collection<br>Current Contents Connect<br>Derwent Innovations Index<br>KCI-Korean Journal Database<br>Medline<br>Russian Science Citation Index<br>SciELO Citation Index	320831
**The Cochrane Central Register of Controlled Trials (CENTRAL)**	
MeSH descriptor: [Kidney Diseases] explode all trees	15687
("kidney disease"):ti,ab,kw	13133
(kidney):ti,ab,kw OR (renal):ti,ab,kw	74738
MeSH descriptor: [Kidney Function Tests] explode all trees	4057
MeSH descriptor: [Glomerular Filtration Rate] explode all trees	2612

(creatinine):ti,ab,kw	25706
(proteinuria):ti,ab,kw OR (microalbuminuria):ti,ab,kw OR (macroalbuminuria):ti,ab,kw	6581
MeSH descriptor: [Renal Insufficiency, Chronic] explode all trees	6493
MeSH descriptor: [Kidney Failure, Chronic] explode all trees	4586
MeSH descriptor: [Uremia] explode all trees	444
(uremia):ti,ab,kw OR (uraemia):ti,ab,kw	895
MeSH descriptor: [Renal Replacement Therapy] explode all trees	8576
MeSH descriptor: [Renal Dialysis] explode all trees	5016
MeSH descriptor: [Hemodiafiltration] explode all trees	236
MeSH descriptor: [Hemodialysis, Home] explode all trees	41
MeSH descriptor: [Peritoneal Dialysis] explode all trees	865
MeSH descriptor: [Peritoneal Dialysis, Continuous Ambulatory] explode all trees	450
(dialysis):ti,ab,kw OR (hemodialysis):ti,ab,kw OR (haemodialysis):ti,ab,kw	17193
(ultrafiltration):ti,ab,kw OR (hemofiltration):ti,ab,kw OR (haemofiltration):ti,ab,kw OR (hemodiafiltration):ti,ab,kw OR (haemodiafiltration):ti,ab,kw	2362
(transplant):ti,ab,kw OR (transplantation):ti,ab,kw OR (transplanted):ti,ab,kw	36559
MeSH descriptor: [Anemia] explode all trees	5154
(anemia):ti,ab,kw OR (anaemia):ti,ab,kw OR (anemic):ti,ab,kw OR (anaemic):ti,ab,kw	19591
MeSH descriptor: [Iron Compounds] explode all trees OR MeSH descriptor: [Ferric Oxide, Saccharated] explode all trees OR MeSH descriptor: [Ferrosoferric Oxide] explode all trees OR MeSH descriptor: [Iron Carbonyl Compounds] explode all trees OR MeSH descriptor: [Iron, Dietary] explode all trees OR MeSH descriptor: [Iron-Dextran Complex] explode all trees	2411
(iron):ti,ab,kw OR (intravenous):ti,ab,kw OR (oral):ti,ab,kw OR ("per os"):ti,ab,kw	255265
MeSH descriptor: [Erythropoietin] explode all trees OR MeSH descriptor: [Epoetin Alfa] explode all trees	2113
(epoetin):ti,ab,kw OR (darbepoetin):ti,ab,kw OR (erythropoietin):ti,ab,kw	5051
(erythropoiesis):ti,ab,kw	1338
MeSH descriptor: [Hemoglobins] explode all trees OR MeSH descriptor: [Transferrins] in all MeSH products OR MeSH descriptor: [Ferritins] explode all trees	10034
(hemoglobin):ti,ab,kw OR (haemoglobin):ti,ab,kw OR (transferrin):ti,ab,kw OR (ferritin):ti,ab,kw	37611
MeSH descriptor: [Clinical Trials as Topic] this term only OR MeSH descriptor: [Controlled Clinical Trials as Topic] this term only OR MeSH descriptor: [Controlled Clinical Trials as Topic] this term only	33350
(trial):ti,ab,kw	815582
MeSH descriptor: [Random Allocation] this term only	20595
(randomized):ti,ab,kw OR (random):ti,ab,kw OR (randomization):ti,ab,kw OR (randomly):ti,ab,kw	958296
MeSH descriptor: [Double-Blind Method] this term only OR MeSH descriptor: [Single-Blind Method] this term only	154632
("double blind"):ti,ab,kw OR ("single blind"):ti,ab,kw OR ("open label"):ti,ab,kw	369653
MeSH descriptor: [Placebos] this term only	23807
(placebo):ti,ab,kw	300326
**ClinicalTrials.gov, the EU Clinical Trials Register, the United Kingdoms' ISRCTN registry**	
kidney disease<br>Also searched for Renal, Renal disease and Nephropathy in ClinicalTrials.gov	8498
creatinine	159
proteinuria	436

microalbuminuria	77
macroalbuminuria	8
uremia<br>Also searched for Uraemia in ClinicalTrials.gov	78
dialysis	1426
hemodialysis<br>Also searched for Haemodialysis and Renal dialysis in ClinicalTrials.gov	1719
ultrafiltration	76
hemofiltration<br>Also searched for Haemofiltration in ClinicalTrials.gov	69
hemodiafiltration<br>Also searched for Haemodiafiltration in ClinicalTrials.gov	92
transplant<br>Also searched for Transplantation and Transplantes in ClinicalTrials.gov	8760
anemia<br>Also searched for Anaemia and Transplantes in ClinicalTrials.gov	3435
anemic<br>Also searched for Anaemic and Transplantes in ClinicalTrials.gov	120
iron	1217
epoetin<br>Also searched for Erythropoietin and Procrit in ClinicalTrials.gov	611
darbepoetin<br>Also searched for Aranesp in ClinicalTrials.gov	165
erythropoiesis	116
hemoglobin<br>Also searched for Haemoglobin in ClinicalTrials.gov	650
transferrin	24
ferritin	61
trial<br>Also searched for Clinical Trials in ClinicalTrials.gov	60470
randomized<br>Also searched for Random in ClinicalTrials.gov	67437
double blind<br>Also searched for Double-Blinded in ClinicalTrials.gov	29196
single blind	3023
open label	24183
placebo<br>Also searched for Placebo-controlled in ClinicalTrials.gov	25751
**DART Europe E-Theses**	
kidney disease	1106
kidney	2520
renal	3256
creatinine	453
proteinuria	264
microalbuminuria	91
macroalbuminuria	9
uremia	47
uraemia	22
dialysis	581
hemodialysis	277

haemodialysis	154
ultrafiltration	300
hemofiltration	16
hemodiafiltration	25
haemodiafiltration	3
transplant	985
transplantation	189
transplanted	691
anemia	504
anaemia	279
anemic	34
anaemic	30
iron	4673
epoetin	15
darbepoetin	14
erythropoietin	295
erythropoiesis	137
hemoglobin	422
transferrin	205
ferritin	178
trial	5245
randomized	2683
random	6642
randomization	203
randomly	2278
double blind	490
single blind	226
open label	134
placebo	1320
**Open Access Theses and Dissertations (OATD)**	
kidney disease	5156
kidney	13171
renal	14203
creatinine	2107
proteinuria	934
microalbuminuria	225
macroalbuminuria	24
uremia	161
uraemia	75
dialysis	3166
hemodialysis	1421
haemodialysis	497
ultrafiltration	1654
hemofiltration	39
haemofiltration	20
hemodiafiltration	57
transplant	5148
transplantation	10494
transplanted	3250
anemia	3729

anaemia	1428
anemic	317
anaemic	192
iron	31275
epoetin	29
darbepoetin	17
erythropoietin	779
erythropoiesis	548
hemoglobin	3684
transferrin	1171
ferritin	954
trial	35049
randomized	20466
random	58349
randomization	1544
randomly	31604
double blind	2805
single blind	1277
open label	683
placebo	7092
**Oral communications and posters presented in relevant medical society meetings**	
Free manual search in<br>American Society of Hematology Annual meeting 2001 to 2019<br>World Congress of the International Society of Hematology 2002, 2005, 2007, 2008, 2010, 2012, 2014, 2016 and 2018<br>American Society of Nephrology (ASN) Kidney Week 2003 to 2019<br>International Society of Nephrology (ISN) World Congress of Nephrology 2001, 2003, 2005, 2007, 2009, 2011, 2013, 2015, 2017 and 2019<br>European Renal Association-European Dialysis and Transplant Association (ERA-EDTA) congress 2003 to 2019<br>World Transplant Congress 2006 and 2014.	100
